# Molecular Docking and Structure-Based Drug Design Strategies

**DOI:** 10.3390/molecules200713384

**Published:** 2015-07-22

**Authors:** Leonardo G. Ferreira, Ricardo N. dos Santos, Glaucius Oliva, Adriano D. Andricopulo

**Affiliations:** Laboratório de Química Medicinal e Computacional, Centro de Pesquisa e Inovação em Biodiversidade e Fármacos, Instituto de Física de São Carlos, Universidade de São Paulo, Av. João Dagnone 1100, São Carlos-SP 13563-120, Brazil; E-Mails: rnsantos@ursa.ifsc.usp.br (R.N.S.); oliva@ifsc.usp.br (G.O.)

**Keywords:** molecular modeling, drug discovery, molecular target, molecular interaction, pharmacophore, virtual screening, SBDD, SBVS

## Abstract

Pharmaceutical research has successfully incorporated a wealth of molecular modeling methods, within a variety of drug discovery programs, to study complex biological and chemical systems. The integration of computational and experimental strategies has been of great value in the identification and development of novel promising compounds. Broadly used in modern drug design, molecular docking methods explore the ligand conformations adopted within the binding sites of macromolecular targets. This approach also estimates the ligand-receptor binding free energy by evaluating critical phenomena involved in the intermolecular recognition process. Today, as a variety of docking algorithms are available, an understanding of the advantages and limitations of each method is of fundamental importance in the development of effective strategies and the generation of relevant results. The purpose of this review is to examine current molecular docking strategies used in drug discovery and medicinal chemistry, exploring the advances in the field and the role played by the integration of structure- and ligand-based methods.

## 1. Introduction

The research-based pharmaceutical industry has increasingly employed modern medicinal chemistry methods, including molecular modeling, as powerful tools for the study of structure-activity relationships (SAR) [1]. In addition to pharmacodynamics data (e.g., potency, affinity, efficacy, selectivity), pharmacokinetic properties (ADMET: absorption, distribution, metabolism, excretion and toxicity) have also been studied through the application of these methodologies [2]. The field has progressed hand-in-hand with advances in biomolecular spectroscopic methods such as X-ray crystallography and nuclear magnetic resonance (NMR), which have enabled striking progress in molecular and structural biology. These techniques have allowed the resolution of more than 100,000 three-dimensional protein structures, providing vital structural information about key macromolecular drug targets [3]. Efforts in storing, organizing and exploring such information have generated a growing demand for robust and sophisticated computational tools. Based on this perspective, the accurate integration of *in silico* and experimental methods has provided the up-to-date understanding of the intricate aspects of intermolecular recognition [4].

Within this framework, structure-based drug design (SBDD) methods (*i.e.*, the use of three-dimensional structural information gathered from biological targets) are a prominent component of modern medicinal chemistry [5]. Molecular docking, structure-based virtual screening (SBVS) and molecular dynamics (MD) are among the most frequently used SBDD strategies due to their wide range of applications in the analysis of molecular recognition events such as binding energetics, molecular interactions and induced conformational changes [6]. A distinct approach in drug design comprises the use of bioactive small-molecule libraries. The unique chemical diversity available in these libraries represents the space occupied by ligands known to interact with a specific target. This type of information is used in ligand-based drug design (LBDD) methods [7]. Ligand-based virtual screening (LBVS), similarity searching, QSAR modeling and pharmacophore generation are some of the most useful LBDD methods [8].

SBDD and LBDD approaches have been applied as valuable drug discovery tools both in academia and industry [9], owing to their versatility and synergistic character. The integration of these approaches has been successfully employed in a number of investigations of structural, chemical and biological data [10,11].

## 2. Structure-Based Drug Design (SBDD)

Understanding the principles by which small-molecule ligands recognize and interact with macromolecules is of great importance in pharmaceutical research and development (R & D) [12]. SBDD refers to the systematic use of structural data (e.g., macromolecular targets, also called receptors), which are usually obtained experimentally or through computational homology modeling [13]. The purpose is to conceive ligands with specific electrostatic and stereochemical attributes to achieve high receptor binding affinity. The availability of three-dimensional macromolecular structures enables a diligent inspection of the binding site topology, including the presence of clefts, cavities and sub-pockets. Electrostatic properties, such as charge distribution, can also be carefully examined. Current SBDD methods allow for the design of ligands containing the necessary features for efficient modulation of the target receptor [12,13]. Selective modulation of a validated drug target by high affinity ligands interferes with specific cellular processes, ultimately leading to the desired pharmacological and therapeutic effects [14].

**Figure 1:**
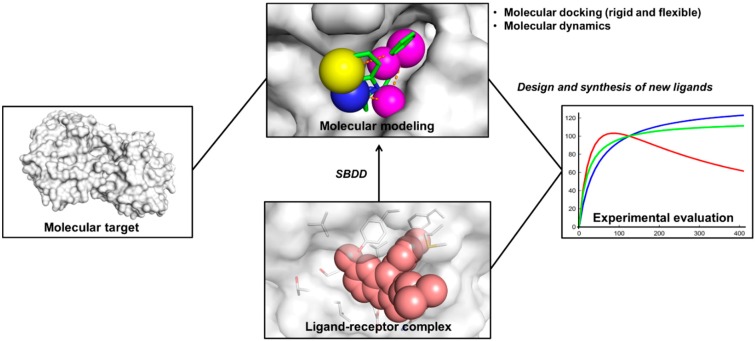
Outline of SBDD. The three-dimensional structure of the molecular target is employed in molecular modeling studies. Promising compounds are synthesized and then experimentally evaluated. Given that bioactive small-molecules are discovered, the structure of a ligand-receptor complex can be obtained. The binding complex is used in molecular modeling studies and novel compounds are designed.

SBDD is a cyclic process consisting of stepwise knowledge acquisition (Figure 1). Starting from a known target structure, *in silico* studies are conducted to identify potential ligands. These molecular modeling procedures are followed by the synthesis of the most promising compounds [15]. Next, evaluations of biological properties, such as potency, affinity and efficacy, are carried out using diverse experimental platforms [16]. Provided that active compounds are identified, the three-dimensional structure of the ligand-receptor complex can be solved. The available structure allows the observation of several intermolecular features supporting the process of molecular recognition. Structural descriptions of ligand-receptor complexes are useful for the investigation of binding conformations, characterization of key intermolecular interactions, characterization of unknown binding sites, mechanistic studies and the elucidation of ligand-induced conformational changes [17].

Once a ligand-receptor complex has been determined, biological activity data are correlated to the structural information [18]. In this way, the SBDD process starts over with new steps to incorporate molecular modifications with the potential to increase the affinity of new ligands for the binding site. The flexibility of the target receptor is an essential aspect that must be considered throughout the modeling phase, bearing in mind that substantial conformational change can occur upon ligand binding. The use of techniques such as flexible docking and MD are useful in addressing the flexibility issue [19,20].

## 3. Molecular Docking

**Figure 2:**
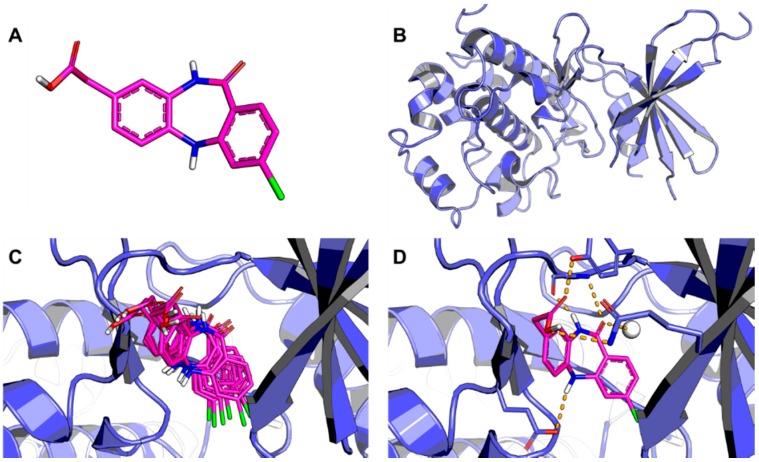
Outline of the molecular docking process. (**A**) Three-dimensional structure of the ligand; (**B**) Three-dimensional structure of the receptor; (**C**) The ligand is docked into the binding cavity of the receptor and the putative conformations are explored; (**D**) The most likely binding conformation and the corresponding intermolecular interactions are identified. The protein backbone is represented as a cartoon. The ligand (carbon in magenta) and active site residues (carbon in blue) are shown in stick representation. Water is shown as a white sphere and hydrogen bonds are indicated as dashed lines.

Molecular docking is one of the most frequently used methods in SBDD because of its ability to predict, with a substantial degree of accuracy, the conformation of small-molecule ligands within the appropriate target binding site (Figure 2) [21]. Following the development of the first algorithms in the 1980s, molecular docking became an essential tool in drug discovery [22]. For example, investigations involving crucial molecular events, including ligand binding modes and the corresponding intermolecular interactions that stabilize the ligand-receptor complex, can be conveniently performed [23]. Furthermore, molecular docking algorithms execute quantitative predictions of binding energetics, providing rankings of docked compounds based on the binding affinity of ligand-receptor complexes [22,23].

The identification of the most likely binding conformations requires two steps: (i) exploration of a large conformational space representing various potential binding modes; (ii) accurate prediction of the interaction energy associated with each of the predicted binding conformations [24]. Molecular docking programs perform these tasks through a cyclical process, in which the ligand conformation is evaluated by specific scoring functions. This process is carried out recursively until converging to a solution of minimum energy [23,24,25].

### 3.1. Conformational Search

**Figure 3:**
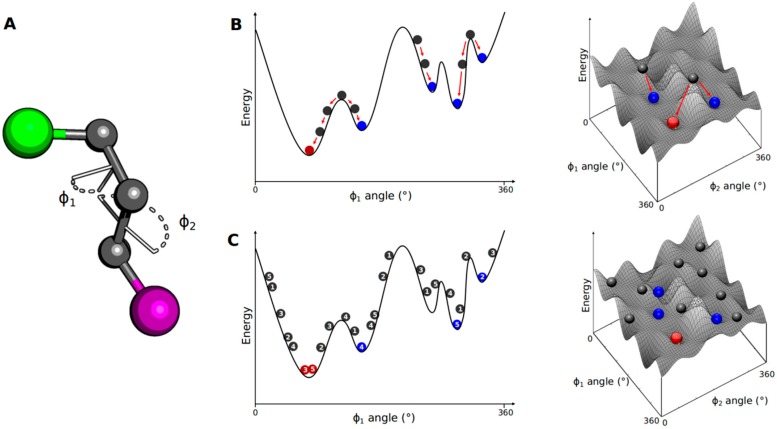
Small-molecule conformational search methods. (**A**) A molecule containing two bulky groups (green and purple spheres) has its conformation defined by two internal dihedrals Φ_1_ and Φ_2_; (**B**) Considering Φ_2_ as a frozen dihedral, the energy variation due to rotation of Φ_1_ is plotted in a 1D energy landscape. The initial structure (grey spheres) is modified by changing Φ_1_, leading to a decrease in energy. The systematic search algorithm changes all structural parameters until a local (blue spheres) or global (red sphere) energy minimum is reached; (**C**) The stochastic search explores the conformational space by randomly generating distinct conformations, populating a broad range of the energy landscape. This procedure increases the probability of finding a global energy minimum.

In the conformational search stage, structural parameters of the ligands, such as torsional (dihedral), translational and rotational degrees of freedom, are incrementally modified (Figure 3A). Conformational search algorithms perform this task by applying systematic and stochastic search methods [25,26].

Systematic search methods promote slight variations in the structural parameters, gradually changing the conformation of the ligands [27]. The algorithm probes the energy landscape of the conformational space and, after numerous search and evaluation cycles, converges to the minimum energy solution corresponding to the most likely binding mode (Figure 3B). Although the method is effective in exploring the conformational space, it can converge to a local minimum rather than the global minimum. This drawback can be overcome by performing simultaneous searches starting from different points of the energy landscape (*i.e.*, distinct conformations) [28].

Stochastic methods carry out the conformational search by randomly modifying the structural parameters of the ligands [29]. For this, the algorithm generates ensembles of molecular conformations and populates a wide range of the energy landscape (Figure 3C). This strategy avoids trapping the final solution at a local energy minimum and increases the probability of finding a global minimum. As the algorithm promotes a broad coverage of the energy landscape, the computational cost associated with this procedure is an important limitation [28,29].

**Figure 4:**
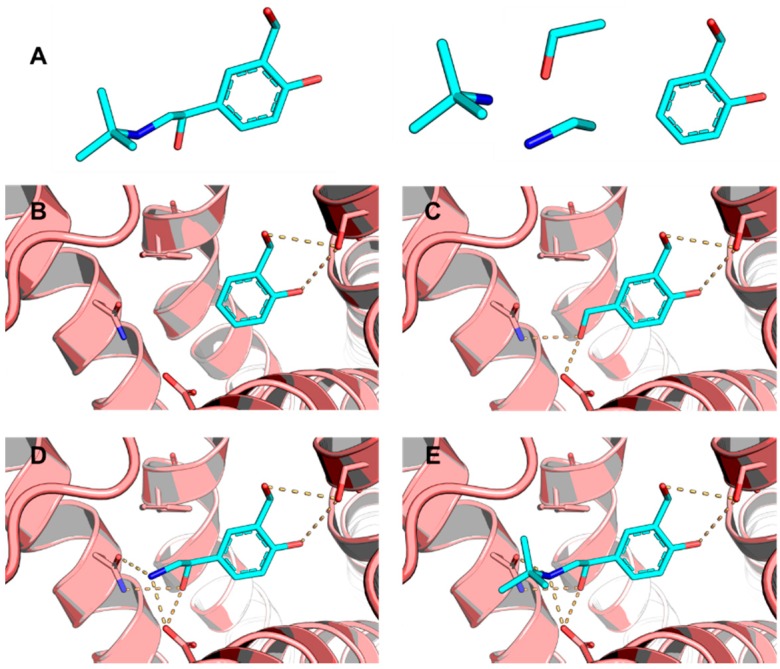
The incremental construction method. (**A**) The ligand (stick representation, carbon in cyan) is broken into several fragments; (**B**) The anchor fragment is docked in the binding site of the molecular target (cartoon representation, carbon in salmon); (**C**) The next fragment is docked after the anchor fragment; (**D** and **E**) The other fragments are docked sequentially to construct the entire ligand in its binding conformation. Residues in the active site are shown in stick representation (carbon in salmon). Hydrogen bonds are indicated as dashed lines.

Systematic and stochastic methods are included in widely used molecular docking programs, which have specific approaches to address their respective problems [27]. For instance, systematic search methods explore all combinations of the structural parameters. The number of possible combinations grows exponentially as the degrees of freedom associated with the ligand increase, resulting in a phenomenon known as combinatorial explosion. Docking programs such as FRED, Surflex and DOCK solve this problem by applying an incremental construction algorithm in which the ligand is gradually built in the binding site (Figure 4) [30,31,32]. In this strategy, the chemical structure is initially broken into several fragments (Figure 4A). Next, one of these parts is selected as an anchor fragment and is docked in a complementary region of the binding site (Figure 4B) while the remaining fragments are sequentially added (Figure 4C–E). The process continues until the entire ligand has been constructed. The algorithm performs the conformational search only for the fragments being added, reducing the degrees of freedom to be explored, and thereby avoiding combinatorial explosion [33].

Genetic algorithms (GA) are an interesting application of the stochastic search, which have been successfully used in molecular docking programs such as AutoDock and Gold [34,35]. The GA algorithm addresses the high computational cost associated with stochastic methods by applying concepts of the theory of evolution and natural selection. As a first step, the algorithm encodes all of the structural parameters of the initial structure in a chromosome, which is represented by a vector. Starting from this chromosome, the random search algorithm generates an initial population of chromosomes covering a wide area of the energy landscape. This population is evaluated and the most adapted chromosomes (*i.e.*, those with the lowest energy values) are selected as templates for the generation of the next population. This procedure decreases the average energy of the chromosome ensemble by transmitting favorable structural characteristics from one population to another, reducing therefore, the conformational space to be explored. The GA routine is recursively executed and, after a reasonable number of conformational search-and-evaluation cycles, converges to a conformation (chromosome) corresponding to the global energy minimum [36].

Regardless the specifics of each method, any conformational search algorithm should be able to explore a wide range of the energy landscape in a reasonable amount of time. Ideally, the evaluation of a modest set of molecules needs to be concluded in a few minutes. A list of widely used molecular docking algorithms categorized according to the conformational search methodology is provided in Table 1.

**Table 1:** Examples of conformational search algorithms.

Systematic Search	Random/Stochastic Search
eHiTS [28]	AutoDock [34]
FRED [30]	Gold [35]
Surflex-Dock [31]	PRO_LEADS [44]
DOCK [32]	EADock [45]
GLIDE [37]	ICM [46]
EUDOC [38]	LigandFit [47]
FlexX [39]	Molegro Virtual Docker [48]
Hammerhead [40]	CDocker [49]
Flog [41]	GlamDock [50]
SLIDE [42]	PLANTS [51]
ADAM [43]	MolDock [52]
	MOE_Dock [53]

### 3.2. Evaluation of Binding Energetics

Molecular docking programs use scoring functions to estimate the binding energetics of the predicted ligand-receptor complexes. The energy variation, due to the formation of the ligand-receptor structure, is given by the binding constant (*K*_d_) and the Gibbs free energy (Δ*G_L_*) [54]. Prediction of the binding energy is performed by evaluating the most important physical-chemical phenomena involved in ligand-receptor binding, including intermolecular interactions, desolvation and entropic effects [55]. Therefore, the greater the number of physical-chemical parameters evaluated, the greater the accuracy of the scoring function. However, the computational cost increases in proportion to the number of variables included in the function, a shortcoming that reduces the productivity of the docking algorithm. Ideally, efficient scoring functions should offer a balance between accuracy and speed, which is a critical aspect when working with large ligand sets.

Scoring functions are categorized in the three following groups: force-field-based, empirical, and knowledge-based functions [56]. Force-field-based scoring functions estimate the binding energy by summing the contributions of bonded (bond stretching, angle bending, and dihedral variation) and non-bonded terms (electrostatic and van der Waals interactions) in a general master function. This type of scoring function applies an *ab initio* method to calculate the energy associated with each term of the function using the equations of classical mechanics [57]. A major limitation of force-field-based methods is their inaccuracy in estimating entropic contributions. This shortcoming is due to the lack of a reasonable physical model to describe this phenomenon. Furthermore, the solvent is not explicitly considered, hindering the estimation of desolvation energies [58].

Empirical scoring functions are another type of evaluation method. Each term of the function describes one type of physical event involved in the formation of the ligand-receptor complex. These include hydrogen-bonding, ionic and apolar interactions, as well as desolvation and entropic effects [59]. As a first step in the development of an empirical function, a series of protein-ligand complexes with known binding affinities is used as a training set to perform a multiple linear regression analysis. Then, the weight constants generated by the statistical model are used as coefficients that adjust the terms of the equation. A drawback of empirical scoring functions is their dependence on the accuracy of the data used to develop the model [60]. However, because of the simplicity of the employed energy terms, empirical functions are faster than force-field-based methods. Surflex and FlexX are broadly used molecular docking programs using empirical scoring functions [31,39].

A third approach used to evaluate ligand-receptor binding energy is the knowledge-based scoring functions. The method uses pairwise energy potentials extracted from known ligand-receptor complexes to obtain a general function [61]. These potentials are constructed by taking into account the frequency with which two different atoms are found within a given distance in the structural dataset. The different types of interactions observed in the dataset are classified and weighted according to their frequency of occurrence. The final score is given as a sum of these individual interactions. As knowledge-based functions do not rely on reproducing binding affinities (empirical methods) or *ab initio* calculations (force-field methods), they offer a suitable balance between accuracy and speed [62].

Every scoring function has its virtues and limitations. Therefore, the simultaneous use of different scoring methodologies has been increasingly employed as a way to obtain a consensus scoring [63]. This can be very useful, as it combines the advantages and simultaneously attenuates the shortcomings of each method [64]. Examples of consensus scoring functions are MultiScore, X-Cscore, GFscore, SCS, SeleX-CS and CONSENSUS-DOCK [65,66,67,68,69,70]. Table 2 provides a list of several scoring functions implemented in the most frequently used molecular docking programs.

Most docking programs are able to successfully predict the conformation of the ligand within the target binding site, as can be confirmed by comparison of predicted complexes with their corresponding crystallographic data. However, most programs do not reproduce the absolute interaction energy of the ligand-receptor complex with satisfactory agreement. Issues such as desolvation and entropic effects are examples of the challenges to be overcome by the current docking algorithms [71,72].

**Table 2:** Examples of scoring functions implemented in widely used molecular docking programs.

Force-Field-Based	Empirical	Knowledge-Based
DOCK [32]	AutoDock [34]	SMoG [82]
AutoDock [34]	GlideScore [37]	DrugScore [62]
GoldScore [35]	ChemScore [60]	PMF_Score [83]
ICM [46]	X_Score [66]	MotifScore [84]
LigandFit [47]	F_Score [73]	RF_Score [85]
Molegro Virtual Docker [48]	Fresno [75]	PESD_SVM [86]
SYBYL_G-Score [73]	SCORE [76]	PoseScore [87]
SYBYL_D-Score [73]	LUDI [77]	
MedusaScore [74]	SFCscore [78]	
	HYDE [79]	
	LigScore [80]	
	PLP [81]	

### 3.3. Covalent Bonds in Molecular Docking

Covalent drugs have demonstrated to be opportune alternatives in several therapeutic areas such as cancer, diabetes, and infectious, cardio-vascular, gastro-intestinal and neurologic diseases. Recent reports have claimed that approximately one-third of the currently marketed enzyme modulators are covalent inhibitors [88]. Covalent ligands act by irreversibly inactivating their targets; consequently, recovery of the inhibited biological function involves re-synthesis of the targeted protein. Usually, covalent inhibitors bind to their molecular targets with high affinity, leading to a long-lasting pharmacological response, and consequently requiring less frequent administration [89]. Well-known drawbacks of covalent drugs such as toxicity, lack of specificity and high reactivity, have led most R & D programs to avoid such compounds [90]. This conception has been reconsidered and an increased interest in covalent inhibitors has been reported recently. As a result, diverse strategies have been developed to approach the binding of covalent small-molecule inhibitors. Covalent docking algorithms are aimed to explore the energy landscape available to the ligand when it is covalently linked to the receptor, as well as evaluate the binding energetics of the interaction [91]. Despite the recent resurgence of covalent drugs, molecular modeling methods devised to address the problem of covalent docking are not as developed as those dedicated to noncovalent docking [92].

Binding of covalent drugs has some differences from noncovalent molecular interaction, especially with respect to binding thermodynamics. Current molecular mechanics (MM) algorithms are able to predict with good accuracy noncovalent binding events. However, the formation of covalent bonds is not satisfactorily approached by these methods [93]. The issue of covalent-bond formation can be appropriately handled by quantum mechanical methods (QM), which are able to explore the whole reaction mechanism [92].

The problem of modeling covalent bonds in molecular docking has been targeted by widely used molecular docking programs such as DOCK [32], AutoDock [34] and Gold [35]. Each of these programs employs a particular approach to manage covalent docking. Gold, for instance, attempts to mimic the covalent bond formation by defining an atom in both the ligand and the receptor to play the role of “link atoms” [94]. Subsequently, the ligand link atom is overlaid on the protein link atom and the geometry of the covalent bond is evaluated by specific terms of the scoring function (clash, torsion and valence-angle bending terms). Another program—DOCKovalent—is an adaptation of DOCK3.6 aimed to perform large-scale, covalent virtual screening [95]. The algorithm defines *a priori* a covalent attachment point and systematically explores the ligand conformational space around the modeled covalent bond. Each conformation is ranked with the default scoring function implemented in DOCK3.6. Another approach is a recent adaptation of AutoDock4, which proposes the so-called two-point attractor method for covalent docking [96]. The default AutoDock routine consists of the calculation of an interaction energy map, constructed by using several probe atoms; and a subsequent conformational search that uses these maps as reference tables to evaluate the binding energetics. The two-point attractor approach works as follows: first, the two terminal atoms of the residue covalently bound to the ligand are removed. Next, this fragment is attached to the correct atom of the ligand, and labeled with two specific atoms types (A and B). Then, a Gaussian function is employed to generate modified interaction maps for these atoms, centered on their original location in the covalently bound amino acid residue. These interaction energy maps penalize ligand conformations in which A or B are not properly placed in their original positions.

### 3.4. Molecular Dynamics

Flexibility of the target binding site is an essential but frequently overlooked aspect to be considered in molecular docking. Enzymes and receptors can undergo conformational changes during the molecular recognition process [97]. In some cases these structural rearrangements are small and the ligand fits in a binding site with little mobility. Otherwise, some proteins undertake significant conformational changes, which can involve elements of secondary and tertiary structure. Such flexibility issues can be handled by the use of techniques such as MD [98].

Usually the ligand stabilizes a subset of several possible conformations of the receptor, shifting the equilibrium toward the minimum energy structures [99]. In such cases, MD simulations can produce alternative conformational states corresponding to these ligand-induced structures. Also, when no suitable crystallographic structures for a particular molecular target are available (*i.e.*, structures with inaccessible or poorly defined binding sites), MD can be applied to generate a set of docking convenient structures [100]. Accordingly, potential conformational states are sampled by MD simulations based on the available crystallographic data, and accessible conformations (*i.e.*, those with accessible and well-defined binding cavities) can be selected for molecular docking [98]. MD can additionally be used to estimate the stability of a ligand-receptor complex proposed by molecular docking [101]. When a MD-generated ligand conformation deviates by more than a given RMSD value from the corresponding docking solution, the predicted ligand-receptor complex can be considered unstable [102].

Molecular dynamics applies Newton’s equations of motion, as described in classical mechanics, to specify the position and speed of each atom in the system under study. As a result, the trajectory and temporal evolution of a ligand-receptor complex can be examined [103]. Initially, a specific configuration is attributed to the atoms with the purpose to reproduce the temperature and pressure of the real system. From the computation of the forces acting on each particle, it is possible to determine the position and velocity of each of these atoms at a posterior time. These calculations are repeatedly performed until the molecular trajectories are integrated for a given time interval [98].

The forces acting on the system are determined by molecular interaction potentials, which are usually parameterized by quantum chemical calculations or experimental data. This set of parameters (the force-field), determines the contribution of each type of interaction to the general function [100]. Among the diverse available force-fields, AMBER [104], CHARMM [105] and GROMOS [106], can be highlighted as widely used in molecular dynamics simulations.

Regardless of its usefulness MD has its limitations. Among them, we can stress the high computational cost demanded by the simulation of large systems, which usually consist of thousands of atoms when ligand-receptor complexes are under study. Some of the conformational changes undertaken by receptors during molecular recognition occur on time scales exceeding the available computational capacity [99]. Despite its limitations, MD is able to deliver important contributions to SBDD, especially when combined with other molecular modeling methods, such as molecular docking.

### 3.5. Structural Water

Crystallographic water is a major challenge in molecular docking and SBDD. These molecules are strongly bound to the receptor and observed across several crystallographic structures of a particular protein [107]. In approximately 65% of the crystallographic protein-ligand complexes, at least one water molecule is involved in ligand-receptor recognition [108]. Usually, structural water is located in deep pockets of the receptor structure and mediates multiple hydrogen bonds between the ligand and the protein binding site [109]. In SBDD and docking campaigns, these molecules can be displaced by the designed ligands or considered as part of the target structure. The release of a crystallographic water molecule from its binding site is entropically favorable; however the process causes a simultaneous loss in enthalpy [109]. To compensate for this enthalpy loss, a specific moiety of the ligand can be designed to mimic the interaction network of the displaced water through the formation of equivalent hydrogen bonds with the protein. Alternatively, structural water can be explicitly included in the docking experiments, allowing the formation of highly favorable hydrogen-bonding networks between the ligand and the target binding site. In this case, a variety of methods are available to evaluate which water molecules are strongly bound and, therefore, suitable for this purpose [107]. Among these strategies one can highlight free energy perturbation calculations using Monte Carlo statistical mechanics simulations, which estimate the binding free energy for a given water molecule, allowing the discrimination between displaceable and strongly-bound structural water [110]. Another strategy is the analysis of geometric parameters of the protein environment surrounding each crystallographic water molecule [111]. One of these methods combines the HINT free energy scoring function [112] and the Rank algorithm, which is a routine that estimates the number and quality of potential hydrogen bonds for a given water molecule [113]. HINT calculates the interaction energy between each crystallographic water molecule and its environment taking into account the chemical properties and accessibility of available hydrogen bond donors and acceptors, as well as the hydrophobic features of the binding site. Rank searches for potential donor and acceptor partners for each water molecule and generates a ranking scheme, ranging from molecules that do not interact via hydrogen-bonding with a non-water element, to molecules that form four geometrically ideal hydrogen bonds. This approach can be used to guide the choice between the replacement of an optimally coordinated structural water molecule—which would require a precisely designed ligand to compensate for the enthalpy loss—or the maintenance of such molecule in the SBDD and docking strategies. Alongside the use of such algorithms, a comparison between structural water in multiple crystal structures should be carried out to minimize the risk of mistakenly discarding or keeping a specific water molecule [109] (strongly bound water is often conserved across multiple crystallographic structures).

### 3.6. Protein-Protein Interaction Inhibitors and Molecular Docking

Cellular and biochemical processes are largely controlled by interactions between different classes of proteins [114]. Many diseases, such as cancer, can be attributed to defective protein-protein interactions (PPIs); therefore, this type of intermolecular event is a highly attractive target in drug discovery [115]. PPI inhibitors can be defined as small-molecule compounds that directly compete with one of the protein partners [116]. The belief that targeting PPIs is an unsuitable strategy in drug design has been challenged by recent successful cases, such as the development AMG-232, a MDM2-p53 inhibitor, currently in Phase II clinical trials for cancer therapy [117]; and maraviroc (Selzentry^®^), a commercially available CCR5-gp120 inhibitor used in AIDS treatment [118].

The major challenge faced by SBDD and molecular docking in the field of PPIs is the identification and characterization of binding sites, as well as the assessment of their potential for interacting with small-molecule compounds [119]. The contact surfaces where two proteins interact with each other are significantly different from ligand-protein binding cavities. Generally, PPI binding sites are relatively flat interfaces that do not have a single large and well-defined pocket; otherwise they consist of a greater number of smaller pockets [119]. Strategies for detection of such binding sites and evaluation of their drugability have been implemented in several computational methodologies, among which one can highlight the freely available web-based tools Q-SiteFinder [120] and ANCHOR [121].

Q-SiteFinder is an energy-based method for the prediction of protein binding sites, found on the assumption that the interaction energy (estimated by a methyl probe) of each interface pocket determines its amenability to SBDD [120]. The distinct pockets at the interface are categorized according to their interaction energy, providing an evaluation of those regions where a ligand could interact and optimize the binding energy. According to this procedure the algorithm generates a volume envelope, which is a region where the calculated van der Waals interaction energy remains below a defined threshold. These volume envelopes indicate the pockets where a putative ligand would engender a favorable interaction with the receptor surface.

The ANCHOR algorithm searches for amino acid side chains deeply buried at protein-protein interfaces (anchor residues) to pinpoint potential binding pockets carrying drugable properties. These anchor sites have the ability to generate a vigorous attraction between the receptor and the ligand, and unlike hotspots, they carry explicit concave/convex surfaces, which is a convenient property for ligand binding. For a particular PPI interface, the method calculates the change in the solvent accessible surface area (ΔSASA) upon binding, for each amino acid side-chain, as well as a measure of their contribution to the total interaction free energy [122]. ANCHOR does not categorize the probed residues as anchor or non-anchor; alternatively it provides a ranking of these residues in decreasing order of ΔSASA. The top ranked residues may be visualized with the assistance of a web-based tool, which offers an interactive interface for visualization of the properties of the surrounding environment, such as the presence of hydrogen-bonding networks.

A distinct strategy is the use of biochemical and biophysical data to guide the docking process. Information provided by biomolecular NMR spectroscopy has been used to define PPI interfaces and determine the reciprocal orientation of the two binding partners [123]. One of these methods, the HADDOCK approach, employs chemical shift perturbation data produced by NMR titration assays as well as information obtained from mutagenesis experiments, to identify the interacting residues and score the predicted binding conformations [124]. Highly solvent-accessible residues that exhibit a substantial chemical shift perturbation upon binding, are labeled as important residues in the formation of the complex. This information is incorporated into the docking procedure as ambiguous interaction restraints (AIR), which take into account the distance between the interacting atoms of the two binding partners. Through this approach, HADDOCK explores all potential configurations around the PPI interface and finds the most favorable pairs of interacting atoms. The method allows side chain flexibility at the PPI interface, which is a key aspect to improve the accuracy of the results. Another useful feature of the HADDOCK methodology is a final refinement step, in which explicit interfacial water molecules are incorporated into the docking protocol to optimize the binding energetics.

## 4. Virtual Screening (VS)

**Figure 5:**
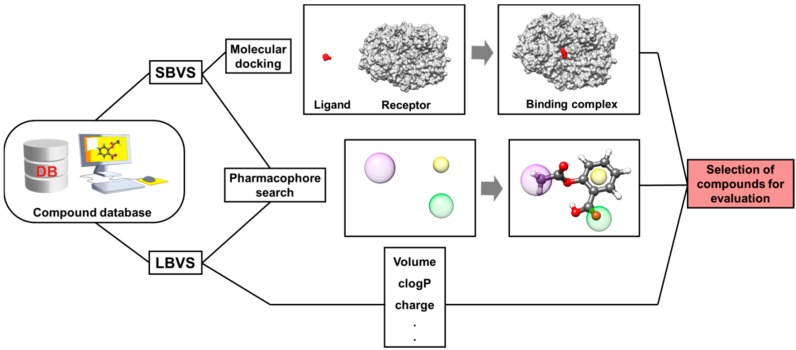
The SBVS and LBVS approaches. Virtual compound databases can undergo different filtering procedures. In SBVS approaches, the three-dimensional structure of the molecular target is employed to identify compounds compatible with the properties of the target binding site. In pharmacophore modeling, compound collections are employed to generate structural patterns that should be present in active compounds. In LBVS studies, molecular descriptors known to be relevant for biological activity are used as selection criteria to identify suitable compounds for experimental evaluation.

Virtual screening is the application of *in silico* methods for selecting promising compounds from chemical databases [125]. It can be regarded as the computational counterpart of experimental biological evaluation methods, such as high-throughput screening (HTS) [126]. In drug discovery, the use of large and chemically diverse compound libraries for computational and biological screening is one of the most widespread strategies [127]. This has stimulated the use of VS as a fast and cost-effective method for the evaluation of a variety of compound collections. Usually, VS strategies fall into two main types: (i) ligand- and (ii) structure-based virtual screening (LBVS and SBVS, respectively, Figure 5) [128].

### 4.1. Ligand-Based Virtual Screening (LBVS)

Although this review focuses on structure-based methods, it is worth mentioning that most of the current virtual screening studies combine SBVS and LBVS methods. A number of reviews on LBVS methods are available in the literature [129,130].

LBVS is based on the exploration of molecular descriptors gathered from compounds known to be active [129,131]. In general, a set of mutual characteristics of a compound series is identified, which are subsequently applied as molecular filters. These database filtering methods are used to select compounds for experimental evaluation and reduce the chemical space to be explored in further screening steps. Several freely available software packages accurately estimate molecular descriptors for database filtering. These programs are useful in predicting important properties related to biological activity, such as solubility, protonation state and molecular volume [132]. Table 3 lists some chemoinformatics tools used to estimate relevant molecular properties.

**Table 3:** Widely used software packages for the prediction of molecular descriptors related to biological activity.

Software
Molinspiration [133,134]
OSIRIS Property Explorer [134,135]
Molsoft [136]
MoKa [137,138]

Another LBVS approach is the use of structural features collected from known ligands to generate pharmacophore models [139]. These ligand-based 3D pharmacophore models consist of a compilation of structural properties thought to be relevant for biological activity. Generating a 3D pharmacophore model involves the following typical steps: (i) exploring the conformational space of the compound series; (ii) identifying reciprocal properties; (iii) aligning the molecules according to the identified properties; and (iv) generating the pharmacophore model. A critical issue that needs to be addressed by current pharmacophore construction algorithms is the development of an effective molecular alignment tool [140]. Table 4 lists several commercial and academic pharmacophore construction programs.

**Table 4:** Examples of programs for pharmacophore generation.

Commercial	Academic
Hiphop [141]	PharmaGist [154,155]
HypoGen [141]	ALADDIN [156]
HypoRefine [141]	RAPID [157]
GASP [142,143]	DANTE [158]
DiscoTech [143,144]	APOLLO [159]
GALAHAD [143,145]	CLEW [160]
LigandScout [146,147]	MPHIL [161]
MOE [148,149]	GAMMA [162]
PHASE [150,151]	SCAMPI [163]
XED [152,153]	Apex-3D [164] LigBuilder [165,166]

### 4.2. Structure-Based Virtual Screening (SBVS)

In SBVS, the compound database is docked into a previously selected target binding site [167]. Along with the prediction of the binding mode, SBVS provides a ranking of the docked molecules. This ranking can be used as the sole criterion for selecting promising molecules, or it can be combined with other evaluation methods. The selected compounds are experimentally evaluated to determine their biological activity on the molecular target under investigation [168].

In general, SBVS consists of the following steps: (i) molecular target preparation; (ii) compound database selection; (iii) molecular docking; and (iv) post-docking analysis. Rigorous review of the available information regarding the target and known ligands, as well as a careful analysis of the advantages and pitfalls of the selected docking algorithms, are required in delineating the most appropriate strategies [169].

It is generally the case that several structures of a given receptor are available. If the target is available in either apo and holo forms, both should be considered in the SBVS strategy. Conformational changes, resulting from interaction with ligands, and structural resolution are important details requiring consideration in the selection of the most suitable structure [170,171]. Next, the selected structure undergoes several procedures to properly prepare it for the molecular docking studies. In short, the preparation routine consists of adding hydrogen atoms, eliminating water molecules (with the exception of those mediating important interactions), specifying the correct protonation and tautomerization states of the binding site residues, and calculating partial charges [172].

Another important step is the preparation of the small-molecule compound collection. Databases that gather, into a single site, a large number of chemical suppliers and a wide diversity of chemical data are widely used in SBVS [173]. Usually, these compound collections operate as interactive interfaces for searching and selecting compound subsets according to predetermined chemical filters. Usually, database compounds are stored as line notations, such as SMILES, SMARTS and InChI, which are then converted into three-dimensional molecular structures. Converting the original files requires the correct assignment of stereochemistry, partial charges, and ionization states [174]. Table 5 provides some examples of well-established virtual databases used in virtual screening.

**Table 5:** Chemical databases used in virtual screening.

Database
Zinc [175,176]
PubChem [177,178]
ChemSpider [179,180]
ChEMBL [181,182]
NuBBE DB [183,184]
ChemBank [185,186]
eMolecules [187]
DrugBank [188,189]
Binding DB [190,191]

In a following step, the prepared database is docked into the target binding site. The conformational search algorithm explores the energy landscape of each molecule and high-scoring compounds are selected as potential ligands [169,170,171,172]. As virtual screening involves hundreds of thousands (or millions) of compounds, post-docking analysis is usually conducted to decide which compounds to prioritize. Visualizing the predicted ligand-receptor complexes is very useful for this purpose, allowing the analyses of critical aspects, such as the presence of specific intermolecular interactions [192]. Another facet that can be assessed through visualization of the predicted structures is whether the solutions match predetermined requirements, such as pharmacophore hypotheses. A list of molecular modeling programs used for visualizing SBVS results is provided in Table 6.

**Table 6:** Programs for graphical display of SBVS and molecular docking results.

Program
UCSF Chimera [193,194]
VMD [195,196]
Pymol [197,198]
BALL [199,200]
RasMol [201,202]
Jmol [203]
JSmol [204]

## 5. Molecular Docking and Structure-Based Drug Design Studies

Molecular docking is a well-established and widely used methodology in drug design. A substantial number of studies are available in which a diverse array of approaches has been applied for the discovery of novel bioactive molecules. Recent cases involving different docking strategies combined with other molecular modeling methods are examined next.

### 5.1. Discovery of Mycobacterium tuberculosis InhA Inhibitors Using SBVS and Pharmacophore Modeling

**Figure 6:**
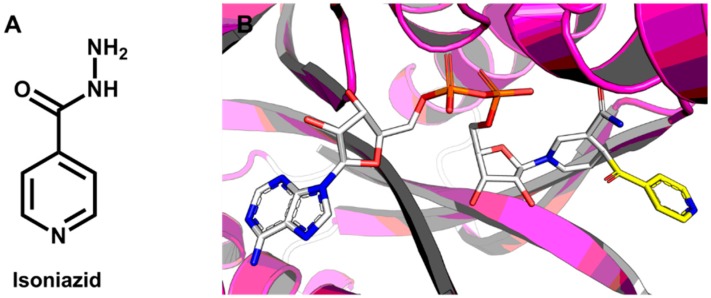
(**A**) Structure of the tuberculostatic drug isoniazid; (**B**) The isonicotinic-acyl moiety covalently bound to NADH in the binding site of InhA (PDB 1ZID, 2.70 Å). The protein backbone is represented as a cartoon. The isonicotinic-acyl fragment (carbon in yellow) and NADH (carbon in white) are shown as sticks.

Trans-enoyl-ACP reductases are NADH-dependent enzymes involved in fatty acid biosynthesis. The enzyme from *Mycobacterium tuberculosis* (InhA) promotes the synthesis of long-chain fatty acids, namely mycolic acids, which are an essential component of the bacterial cell wall [205]. An important enzyme in tuberculosis drug discovery, InhA is the molecular target for the tuberculostatic drug isoniazid [206]. The compound is a pro-drug that loses its hydrazine group as it reacts with the enzyme cofactor NADH, forming an isonicotinic-acyl-NADH complex that inhibits the catalytic activity of InhA (Figure 6).

**Figure 7:**
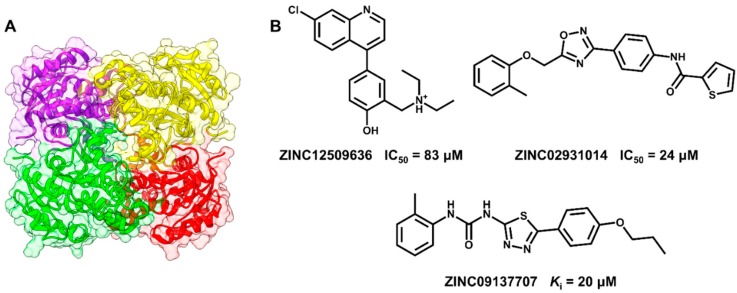
(**A**) The crystallographic structure of the InhA homotetramer (PDB 1P44, 2.70 Å); (**B**) The structure and activity of the InhA inhibitors identified using the pharmacophore modeling and SBVS approaches.

In a recent study, a series of novel InhA inhibitors was identified through a virtual screening strategy. The authors employed a multistage approach, integrating pharmacophore modeling and molecular docking [207]. First, a four-point 3D pharmacophore model was generated by superimposing 36 InhA crystallographic structures available in the PDB. Next, an analysis of the enzyme binding site (PDB 1P44, 2.70 Å) (Figure 7A) was conducted to identify a set of structural properties for use as molecular filters. According to this analysis, the ZINC database was filtered by applying the following rules: 4 < log*P* < 7; number of rotatable bonds < 6; number of hydrogen bond acceptors < 8; polar surface area < 40 Å^2^; 250 Da < molecular weight < 400 Da. The resulting dataset containing 999,853 molecules underwent two screening procedures: (i) a pharmacophore-based VS; and (ii) a SBVS employing four molecular docking programs.

The filtered database was subjected to a flexible search using the 3D pharmacophore model and the database searching program UNITY [208]. A group of compounds matching the pharmacophore hypothesis (*i.e.*, those assigned with the highest UNITY scores) was docked into the InhA binding site using the molecular docking program Gold. This program was chosen because it was able to predict the crystallographic poses of known ligands with high accuracy. The solutions were visually inspected and those molecules matching all of the four pharmacophore spots were selected for further analysis.

In parallel with the pharmacophore-based approach, the database (999,853 compounds) was docked into the InhA binding site using Gold. The 100 top scored compounds were subjected to a SBVS using three other molecular docking programs (AutoDock, FlexX and Surflex-Dock) to acquire a consensus score.

After a detailed analysis of the results obtained with both strategies (pharmacophore-based and structure-based VS), six ligands were evaluated for their activity on purified InhA. Three molecules, having IC_50_ and *K*_i_ values in the micromolar range, were identified as suitable starting points for further ligand optimization studies in tuberculosis drug discovery (Figure 7B).

### 5.2. Discovery of Proteasome Inhibitors by SBVS

**Figure 8:**
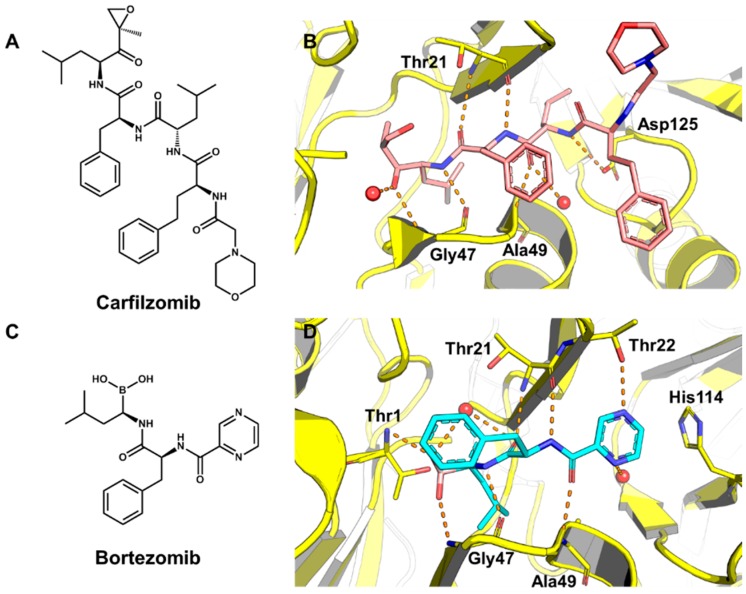
(**A**) Structure of the proteasome inhibitor carfilzomib; (**B**) Crystallographic structure of the human 20S proteasome complexed to carfilzomib (PDB 4R67, 2.89 Å). The protein backbone is in cartoon representation. The ligand (carbon in salmon) and residues in the active site (carbon in yellow) are shown in stick representation. Water is shown as red spheres and hydrogen bonds are indicated as dashed lines; (**C**) Structure of the proteasome inhibitor bortezomib; (**D**) Crystallographic structure of a yeast 20S proteasome complexed to bortezomib (PDB 2F16, 2.80 Å). The ligand (carbon in cyan) is shown in stick representation.

The proteasome plays an essential role in eukaryotic metabolism by promoting the degradation of damaged and unnecessary proteins [209]. This protein complex also controls the progression of the cell cycle, an aspect that led to its development and validation as a molecular target for cancer drug discovery. Two proteasome inhibitors, carfilzomib, a peptidic natural product derivative, and bortezomib, a boronic acid-based compound, have been successfully used in the treatment of refractory multiple myeloma. The discovery and development of such chemotherapeutic agents represented a significant advance in the therapy of the disease [210]. Figure 8 depicts the structure of carfilzomib and bortezomib and their crystallographic structures in complex with the 20S proteasome.

A SBVS strategy was employed to identify novel proteasome inhibitors from an *in-house* database containing 345,000 compounds at the University of Cincinnati [210]. This compound collection was evaluated by applying a rigid molecular docking algorithm implemented in the FRED program. The molecules were ranked through consensus scoring using distinct force-field-based functions and the most highly scored compounds were additionally checked for specific binding conformations. Finally, 288 molecules were selected and evaluated for their activity on purified human 20S proteasome.

**Figure 9:**
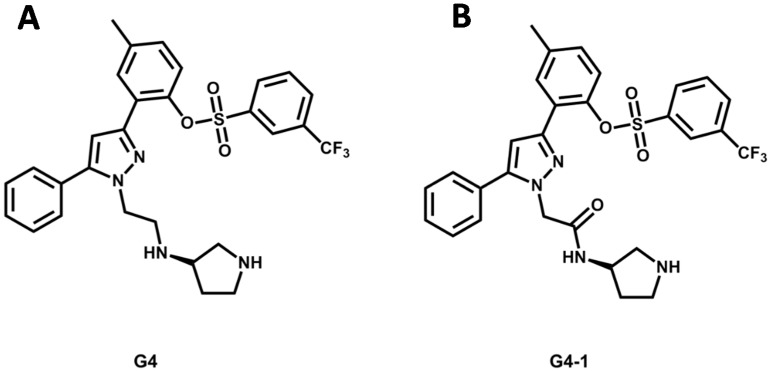
Structures of G4 (**A**) and G4-1 (**B**), the most potent proteasome inhibitors identified by SBVS. Remaining CT-L (chymotrypsin-like) activity at 10 μM: 9.69% for G4 and 6.15% for G4-1.

Of the 288 tested compounds, 19 were found to be active as proteasome inhibitors. These results led to further studies in which the inhibitor G4 (Figure 9A) emerged as a promising molecule capable of inhibiting the replication of cancer cells *in vitro* (IC_50_ = 7 μM). Compound G4 was also used as a reference structure from which a series of derivatives was designed. This approach resulted in the identification of compound G4-1 (Figure 9B) as a lead proteasome inhibitor with attractive drug-like properties. This compound stands out for its excellent metabolic stability compared to the currently marketed proteasome inhibitors carfilzomib and bortezomib.

### 5.3. Identification of a New Series of STAT3 Inhibitors by VS

Signal transducers and transcription activators (STATs) are transcription factors activated by phosphorylation and dimerization. STAT dimers bind to recognition motifs in DNA, thereby promoting gene transcription [211]. This event triggers signal transduction pathways that regulate many biological processes including cell survival, growth and differentiation. Among the seven STAT isoforms, STAT3 has been investigated as a molecular target for cancer drug discovery. In neoplastic cells, STAT3 disarticulates the correct expression of transcription factors, cell cycle regulators and angiogenesis factors, inducing the transformation of benign tumors into malignant cells [212].

**Figure 10:**
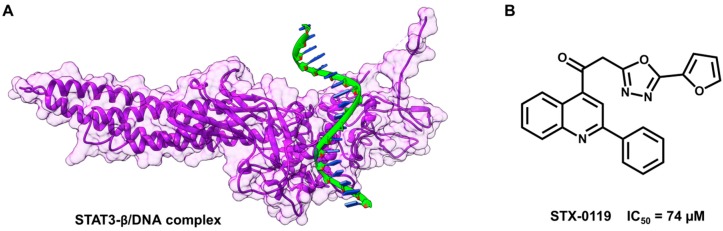
(**A**) Structure of STAT3-β in complex with DNA (PDB 1BG1, 2.25 Å). The protein backbone is in cartoon representation (purple). The DNA strand is shown in green (deoxyribose), blue (purine and pyrimidine bases) and red (phosphates); (**B**) Structure of the STAT3 dimerization inhibitor STX-0119 identified by molecular docking studies.

Recently, a series of novel STAT3 dimerization inhibitors was discovered through an SBVS campaign [213]. A database containing millions of compounds was filtered for drug-like properties, resulting in a final collection of approximately 360,000 molecules, which was then used in the SBVS. The crystallographic structure of the STAT3-β dimer (PDB 1BG1, 2.25 Å) (Figure 10A) was elected as the receptor for the docking studies. The most promising compounds were selected by applying a consensus scoring method called CONSENSUS-DOCK, implemented in the molecular docking program DOCK4. This methodology estimates the energetics of ligand-receptor binding by combining three scoring functions: DOCK4, FlexX and PMF.

Consensus scoring and visual inspection of the ligand-receptor complexes were used for the selection of potential inhibitors. Through this process, 136 molecules were evaluated for their ability to inhibit STAT3 dimerization. Compound STX-0119, a quinolone-carboxamide derivative (Figure 10B), was the most active STAT3 dimerization inhibitor. Studies using fluorescence resonance energy transfer (FRET) assays confirmed that STX-0119 acts by disrupting STAT3 dimerization. *In vivo* studies in mice models showed the ability of the compound in suppressing the replication of lymphoma cells, indicating the suitability of STX-0119 for further medicinal chemistry studies.

### 5.4. Discovery of Pim-1 Kinase Inhibitors by a Hierarchical Multistage VS

Serine-threonine kinase proviral insertions in murine (Pim-1) plays an important role in cell proliferation and differentiation. The enzyme promotes cell cycle progression, regulating essential signaling pathways such as janus kinase/signal transducer and activator of transcription (JAK/STAT) [214]. Similarly to other kinases involved in cell cycle regulation, Pim-1 has been studied as a potential molecular target for cancer drug R & D. Abnormal levels of Pim-1 have been correlated to the genesis of hematopoietic and solid tumors such as lymphoblastic leukemia, acute myeloid leukemia, chronic myeloid leukemia, non-Hodgkin’s lymphoma, and prostate and breast cancers [215]. The enzyme has also been implicated in the conversion of benign neoplasms into malignant tumors and in the emergence of drug resistance [215,216].

**Figure 11:**
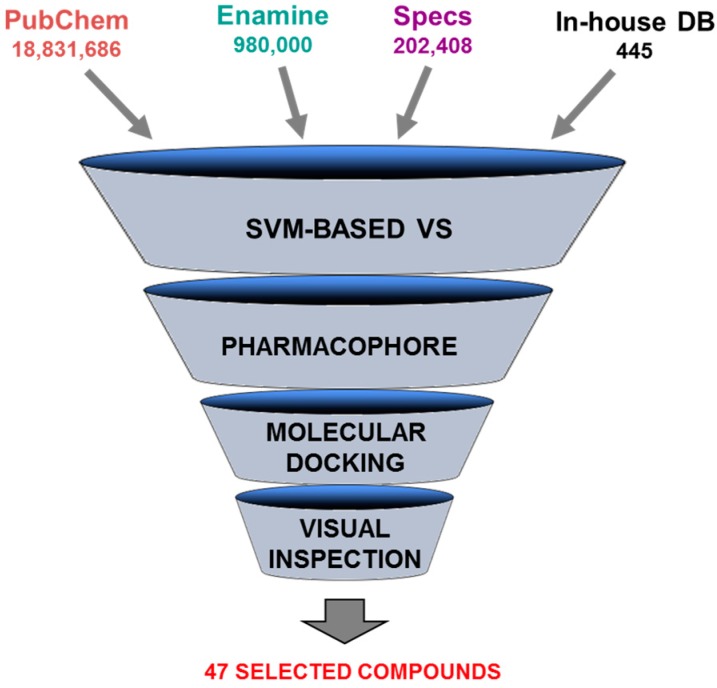
Scheme of the multistage VS approach. Four compound collections were combined yielding a database containing approximately 20 million compounds. Three computational screening procedures were applied and followed by visual inspection. The overall process resulted in the selection of 47 compounds for biochemical evaluation.

Novel Pim-1 inhibitors were discovered by applying a combination of ligand- and structure-based filtering methods [216]. Four compound libraries, approximately 20 million molecules in all, were employed in a hierarchical multistage virtual screening strategy. Three molecular modeling approaches were used sequentially: (i) support vector machine modeling (SVM); (ii) pharmacophore construction; and (iii) molecular docking (Figure 11).

First, a SVM model was built using a training set of almost 37,500 compounds and a group of 50 molecular descriptors including geometrical, topological, and electronic properties. The reliability of the model was confirmed by the attainment of a prediction accuracy of approximately 99% for the training set and 90% for an external test set. This SVM classification model was used as an initial filtering stage wherein the complete database (20 million compounds) was reduced to 56,583 molecules. In the following screening step, a pharmacophore-based strategy was employed. A 3D pharmacophore hypothesis (defined by one hydrogen-bond acceptor, one hydrogen-bond donor and one hydrophobic spot) was built according to eight highly potent Pim-1 inhibitors. This ligand-based pharmacophore model was employed to evaluate the output of the SVM screening, resulting in a subset of 10,631 compounds.

**Figure 12:**
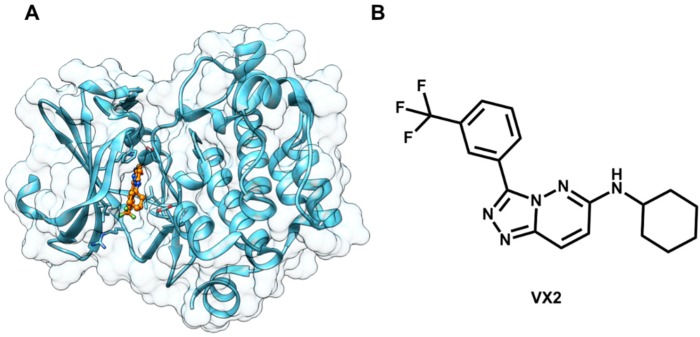
(**A**) Crystallographic structure of Pim-1 kinase complexed to the inhibitor VX2 (PDB 3BGQ, 2.00 Å). The protein backbone is in cartoon representation. The inhibitor (carbon in orange) is shown as ball-and-sticks; (**B**) Structure of the Pim-1 inhibitor VX2.

**Figure 13:**
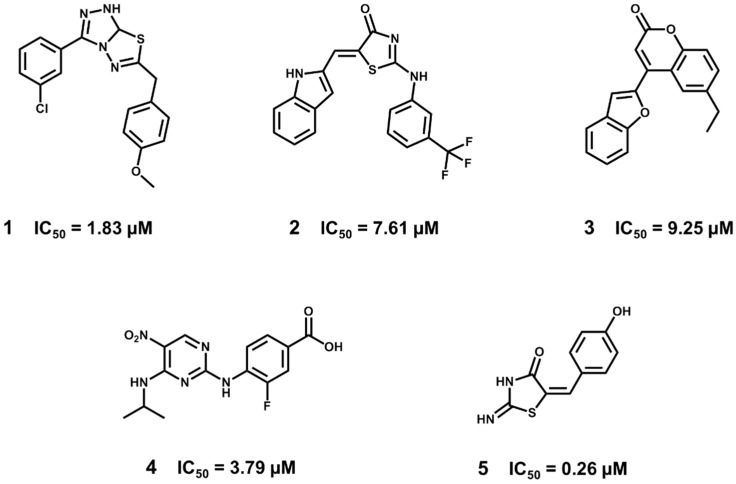
Structures and biological activity data for the Pim-1 inhibitors identified in the ligand- and structure-based VS strategy.

Finally, this enriched database was subjected to a SBVS procedure. The molecular docking program Gold and the crystallographic structure of Pim-1 complexed to a pyridazine inhibitor (PDB 3BGQ, 2.00 Å) (Figure 12) were used in the virtual screening stage. The 935 most highly scored compounds were selected for visual inspection, from which 47 molecules were chosen for *in vitro* biochemical assays. The overall VS strategy identified five compounds as novel Pim-1 inhibitors (Figure 13). The study demonstrated the successful integration of ligand- and structure-based approaches in the discovery of suitable hits for further development of new chemotherapeutic agents.

### 5.5. Identification of Aldose Reductase Inhibitors by MD and SBVS

Human aldose reductase (ALR2) is a NADPH-dependent oxidoreductase that catalyzes the reduction of glucose to sorbitol [217]. This is a key reaction in the polyol pathway of glucose metabolism, a process implicated in the long-term complications of diabetes. Hyperglycemia induces overexpression of ALR2, leading to an increase in sorbitol concentration, which ultimately causes osmotic imbalance, alterations in membrane permeability, oxidative stress, and finally tissue damage [218]. Accordingly, the enzyme has been extensively studied as a molecular target for the treatment of diabetes complications. Several ALR2 inhibitors have entered clinical trials. Currently, epalrestat (Kinedak^®^) is marketed in Japan as an ALR2 inhibitor for the treatment of diabetic neuropathy [219].

**Figure 14:**
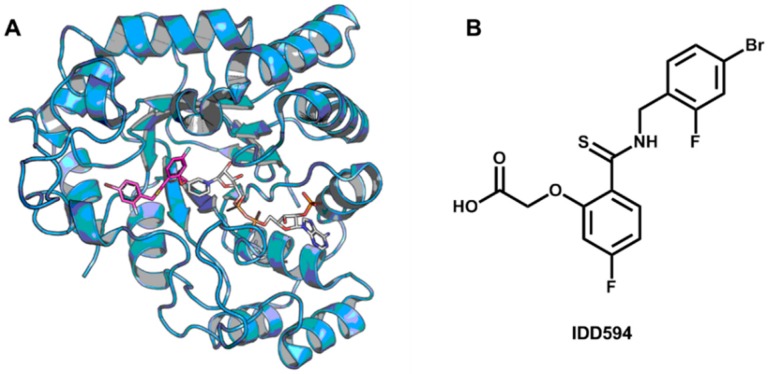
(**A**) Crystallographic structure of ALR2 complexed to the inhibitor IDD594 and the enzyme cofactor NADP^+^ (PDB 1US0, 0.66 Å). The protein backbone is in cartoon representation. The inhibitor (carbon in magenta) and NADP^+^ (carbon in white) are shown as sticks; (**B**) Structure of the ALR2 inhibitor IDD594.

In a recent investigation, novel ALR2 inhibitors were discovered by an approach incorporating MD simulations and SBVS studies [219]. The binding site of the enzyme undergoes large conformational changes and adopts distinct configurations upon binding different classes of ligands. This variability imposes an additional difficulty in the characterization of a unique and well-defined binding site appropriate for molecular docking studies. To address this issue, potentially accessible binding site conformations were sampled by MD simulations based on the available crystallographic structures of ALR2. After this procedure, three average conformations were selected for the SBVS campaign. Figure 14 shows one of the crystallographic structures of ALR2 used in the MD simulations. The enzyme is complexed to the inhibitor IDD594 (PDB 1US0, 0.66 Å).

A database containing more than 7200 compounds was docked against the three representative structures using the molecular docking program FlexX. Approximately 1200 highly scored compounds were retained and subsequently subjected to protein-ligand interaction analysis. This analysis was based on an intermolecular interaction fingerprinting method, by which a set of 128 molecules were selected for the next screening stage. The ligand-receptor complexes of these compounds underwent MD simulations, and 71 molecules with RMSD values less than 3.00 Å from the docking conformation were selected for biochemical evaluation.

**Figure 15:**
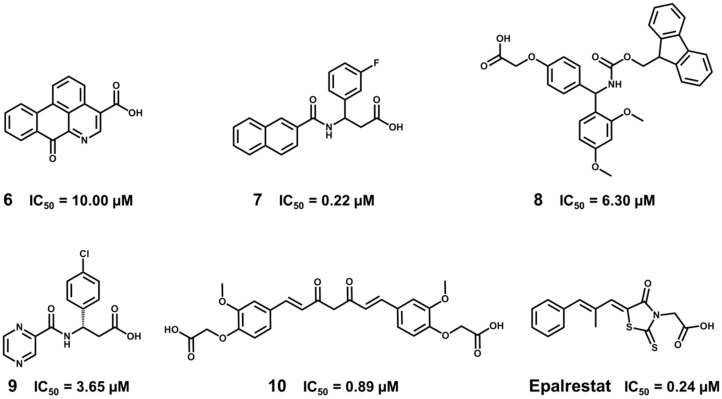
Structures and biological activity data for the ALR2 inhibitors identified by the VS strategy. Epalrestat (Kinedak^®^), a commercially available ALR2 inhibitor is also shown.

Experimental validation revealed 15 novel ALR2 inhibitors having IC_50_ values in the micromolar or nanomolar range (Figure 15). The most potent inhibitors demonstrated biological activity comparable to the marketed ALR2 inhibitor epalrestat (IC_50_ = 0.24 μM). Some of these compounds also showed ALR2/ALR1 selectivity and cytotoxicity comparable to epalrestat. These data demonstrate the value of the discovered compounds, which can be considered as promising candidates for diabetes drug discovery.

### 5.6. Design of Selective Cyclooxygenase-2 Inhibitors

Cyclooxygenase-2 (COX-2) is an enzyme expressed in inflammatory reactions. It converts arachidonic acid into prostaglandin-H2, the early step in the biosynthesis of proinflammatory lipids called prostanoids. The other isoform of the enzyme (COX-1) is constitutively expressed in several tissues and plays a key role in maintaining the homeostasis in normal cells [220].

COX-1 and COX-2 are well-known molecular targets for nonsteroidal anti-inflammatory drugs (NSAIDs), and have been investigated in neuroinflammatory conditions such as Alzheimer’s disease (AD) [221]. The physiopathology of AD involves the accumulation of amyloidogenic Aβ peptides within the brain which leads to mitochondrial dysfunction, oxidative stress, and ultimately to neurodegeneration [222]. Accordingly, inhibition of amyloid deposition and destabilization of amyloid aggregates have been investigated in AD drug discovery [223].

**Figure 16:**
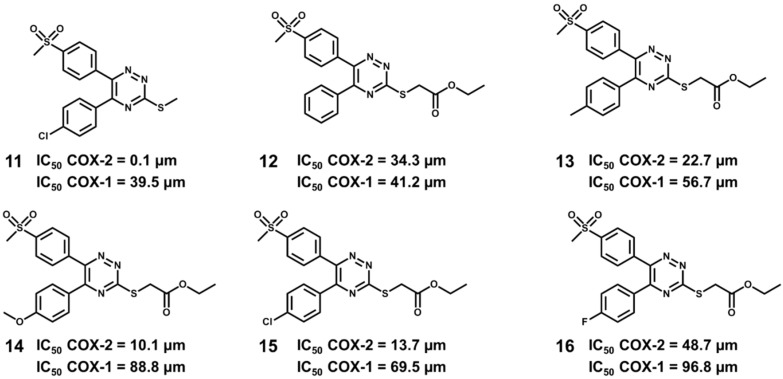
Structure and biological activity of the designed series of COX inhibitors.

**Figure 17:**
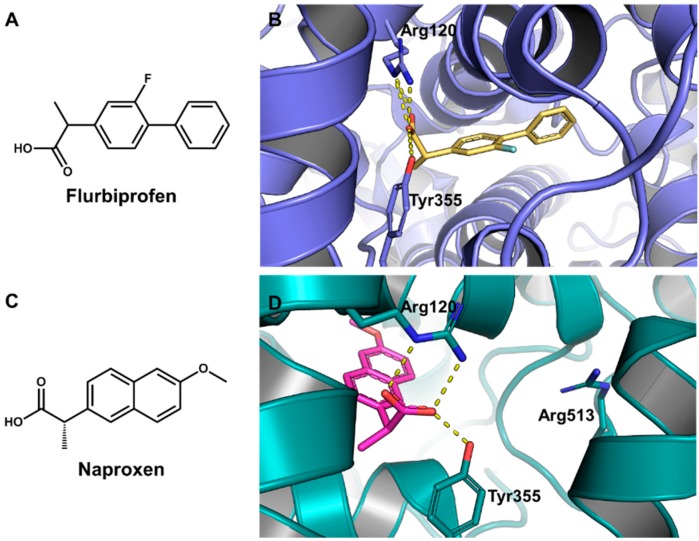
(**A**) Structure of the COX inhibitor flurbiprofen; (**B**) Crystallographic structure of COX-1 complexed to flurbiprofen (PDB 3N8Z, 2.90 Å). The protein backbone is in cartoon representation. The ligand and residues in the active site are shown in stick representation. Hydrogen bonds are indicated as dashed lines; (**C**) Structure of the COX inhibitor naproxen; (**D**) Crystallographic structure of COX-2 complexed to naproxen (PDB 3NT1, 1.73 Å). The protein backbone is in cartoon representation. The ligand and residues in the active are shown in stick representation.

Recently, a series of selective COX-2 inhibitors acting as β-amyloid aggregation blockers was discovered via molecular docking [224]. Based on the structure of a diaryltriazine lead (compound 11, Figure 16), a series of derivatives was designed and docked into the active site of both COX-1 (PDB 3N8Z, 2.90 Å) and COX-2 (PDB 3NT1, 1.73 Å) (Figure 17) using AutoDock4.2. The presence of key molecular interactions and the calculated binding free energy were used to evaluate the reliability of the predicted enzyme-inhibitor complexes. All compounds demonstrated higher affinity for COX-2 than for COX-1, as shown by the Gibbs free energy of binding (−8.66 to −9.49 kcal/mol for COX-2 and −0.65 to −5.25 kcal/mol for COX-1). The inhibitors showed the expected molecular interactions, particularly with the so-called selectivity site, where Arg513 plays a critical role in selective COX-2 inhibition.

Among the designed series, one can highlight compound 14 which showed an IC_50_ value of 10.1 μM in the COX-2 inhibition assay, with a selectivity index of 8.79. Additionally, this inhibitor displayed remarkable *in vitro* disaggregation activity on β-amyloid peptides (94% inhibition for Aβ_1–40_ and 93% for Aβ_1–42_). The ability to selectively inhibit COX-2 while maintaining residual activity on COX-1, along with the potent anti-aggregation activity on Aβ peptides, indicate the suitability of compound 14 as a promising starting point for molecular optimization in AD drug discovery.

## 6. Conclusions

The principles and methods discussed in this review highlight the strategies by which molecular docking and SBDD approaches have been applied in the identification of novel bioactive compounds. Undoubtedly, challenges still remain, especially for issues involving the accuracy of the available scoring functions, which are in fact classical approximations of events ruled by quantum mechanics. Most molecular docking programs successfully predict the binding modes of small-molecule ligands within receptor binding sites. However, the current algorithms do not estimate the absolute energy associated with the intermolecular interaction with satisfactory accuracy. The appropriate handling of issues such as solvent effects, entropic effects, and receptor flexibility are major challenges that require attention. Successful molecular docking protocols require a solid knowledge of the fundamentals of the applied methods. Understanding these principles is essential in the production of meaningful results. Molecular docking has several strengths, among which the method’s ability to screen large compound databases at low cost compared to experimental techniques such as HTS is particularly notable. In the current panorama of drug discovery, where high attrition rates are a major concern, properly designed VS strategies are time-saving, cost-effective and productive alternatives. As shown in the highlighted case studies, molecular docking has been able to identify promising compounds that might represent future solutions in critical areas of human health.
